# Cognitive bias in animal behavior science: a philosophical perspective

**DOI:** 10.1007/s10071-022-01647-z

**Published:** 2022-07-04

**Authors:** Behzad Nematipour, Marko Bračić, Ulrich Krohs

**Affiliations:** 1 Center for Philosophy of Science, University of Münster, Domplatz 23, 48143 Münster, Germany; 2 Department of Behavioural Biology, University of Münster, Badestr. 13, 48149 Münster, Germany; 3 Department of Philosophy, University of Münster, Domplatz 23, 48143 Münster, Germany

**Keywords:** Ambiguous stimuli, Cognitive bias, Judgment bias, Emotions, Representation, Decision-making

## Abstract

Emotional states of animals influence their cognitive processes as well as their behavior. Assessing emotional states is important for animal welfare science as well as for many fields of neuroscience, behavior science, and biomedicine. This can be done in different ways, e.g. through assessing animals’ physiological states or interpreting their behaviors. This paper focuses on the so-called *cognitive judgment bias* test, which has gained special attention in the last 2 decades and has become a highly important tool for measuring emotional states in non-human animals. However, less attention has been given to the epistemology of the cognitive judgment bias test and to disentangling the relevance of different steps in the underlying cognitive mechanisms. This paper sheds some light on both the epistemology of the methods and the architecture of the underlying cognitive abilities of the tested animals. Based on this reconstruction, we propose a scheme for classifying and assessing different cognitive abilities involved in cognitive judgment bias tests.

## Introduction

Assessing animals’ emotional states has explanatory, predictive, and illustrative value for animal welfare science, neuroscience, and psychopharmacology (Mendl et al. 2009), as well as for the discourse concerning attributing rights to *sentient species*. However, this assessment is particularly difficult in non-human animals because of the lack of verbal interaction. Consequently, scientists in these fields are looking for various indicators of emotional states such as behavioral and physiological changes that accompany such states to assess in which emotional state an animal is, or whether or not animals of the considered species experience them at all (Kremer et al. 2020). For example, the state of fear may be accompanied by behavior like freezing, fleeing, or even attacking, and by physiological changes such as increased heart rate, increased blood pressure, and enhanced levels of circulating glucocorticoids (Mendl et al. 2009). In biomedical research, animal models for emotional disorders, such as anxiety and depression, are often based on exposing animals to stressful conditions and then recording behavioral indicators, e.g. immobility, exploration versus avoidance, self-grooming, and vocalizations (see Bourin 2015; Kalueff et al. 2016; Simola and Granon 2019; Wang et al. 2017). Animal research, in general, uses emotional indicators mostly to detect negative emotional states (Proctor et al. 2013) and the methods for assessing positive states are limited (Paul et al. 2005). This limits the research of emotions, particularly from the perspective of animal welfare science, which aims at finding ways to induce positive states, in addition to reducing pain and suffering in animals (Boissy et al. 2007). Additionally, many commonly used indicators suffer from certain epistemic problems (which are discussed in detail in the next section). This motivates scientists to consider novel indicators that are potentially more reliable and can also detect positive states (Kremer et al. 2020).

An increasingly used indicator of emotional states in non-human animals is *cognitive bias* (Paul et al. 2005). This indicator has its background in psychological experiments on humans. Emotional states affect our memory, attention, and judgment (Mathews et al. 1995; Mineka and Sutton, 1992). A paradigmatic example of such influences is that people in negative emotional states, like anxiety, depression, or fear, tend to remember and focus on negative events and interpret ambiguous situations negatively.

The potential utility of testing cognitive bias in welfare research was demonstrated in the seminal study of Harding et al. (2004), who showed that rats housed in “unpredictable”/stressful conditions (which cause depression-like symptoms) were inclined to respond more negatively to ambiguous situations than rats housed in “predictable”/familiar conditions.^1^ Their judgment was biased. The authors suggested using behavioral responses in ambiguous situations as an indicator of emotional states (Harding et al. 2004; Paul et al. 2005), which initiated numerous studies that demonstrated that cognitive judgment bias can be found in a wide range of taxa, from pigs to bumblebees (reviewed in Lagisz et al. 2020; Mendl et al. 2009; Neville et al. 2020). Since both pharmacological and environmental manipulations of affective states alter judgment bias (reviewed in Lagisz et al. 2020; Neville et al. 2020), cognitive judgment bias tests can be considered a promising tool for assessing emotional states of non-human animals.

In this paper, we pursue two main goals. First, we want to examine the epistemic role of judgment bias as an indicator of emotions. We start by pointing out known epistemic problems with the more traditional indicators of emotional states (behavioral and physiological changes) and point out some advantages as well as limits of using judgment bias as an indicator of emotional states in animals. We aim at assessing the epistemic value of the judgment bias test and demonstrate its empirical motivation.

Second, we scrutinize judgment bias as such. What kind of cognitive abilities are in play? We are not engaging, however, in a conceptual analysis of the notion of judgment bias, but rather, looking at cognitive abilities underlying the judgment bias that is used as an indicator of emotional states, and aiming at determining what kind of abilities these are. Animal welfare science may not need to determine exactly what kind of ability underlies an indicator as long as the indicator enables tracking or individuating the emotional states. However, limitations of the judgment bias test could depend on which cognitive ability is in play, so identifying them could assist in improving test designs and interpretation of the results. Additionally, other perspectives identifying underlying cognitive abilities are worth pursuing, because: (1) it could help us to understand how individuals make decisions in ecologically relevant situations (e.g. ambiguous rustle in a bush potentially indicating either a predator or prey); (2) pinpointing underlying cognitive abilities in different species may clarify minimal requirements for cognitive and emotion-like systems to produce such a phenomenon and help us better understand the evolution of higher cognitive abilities; (3) even from the perspective of animal welfare studies, there are disparities between treatments of animals with higher and lower cognitive abilities. Therefore, it may be important to determine which level of cognitive abilities is in play in cases of judgment bias. This is important, because evidence of judgment bias across the animal kingdom has fueled a heated debate on attributing emotional states and consciousness to species that are usually not considered as being sentient (Mendl and Paul 2016)—a debate that has ramifications for questions concerning animal welfare and animal rights.

## Epistemic limits of emotional indicators

Most scientists seem to agree that at least some emotional states can be ascribed to (some) non-human animals (Scarantino et al. 2021). However, in affective science, there are not only many different theories on what constitutes emotions, but the terminology is also used in an inconsistent way, which can create misunderstandings when discussing emotions in non-human animals (see e.g. Adolphs and Andler 2018; Barrett et al. 2007; Izard 2010; LeDoux 2012; Paul and Mendl 2018). Therefore, before discussing the limitations of emotional indicators, we need to clarify the terminology. In this paper, we use the term “emotional state” broadly, referring to inner representational states without presupposing subjective or conscious experience. Concerning the structure of emotional states, we try to generalize across both discrete approaches—considering basic emotions as discrete entities, underlaid by separate neurological systems (see Ekman 1992) and dimensional approaches—specifying emotions by the position in multidimensional space, with two common dimensions being valence (i.e., pleasantness or unpleasantness of emotional state) and arousal (i.e., activity or energy level) (see Russell and Barrett 1999).

Since emotions are not directly observable, assessing emotional states requires the use of indicators. There are two major types of problems with emotional indicators like behavioral and physiological changes. First, change in an indicator does not need to be unique to a specific emotional state. Two or more different emotional states could be accompanied by the same/similar change in a physiological or behavioral measure.^2^ This means that the indicators are not always indicators of *uniquely one* emotional state (e.g. fear) or emotional dimension (e.g. unpleasant). This is problematic in biomedical and animal welfare research when trying to assess whether an animal is in a specific emotional state. For example, detection of an elevated level of circulating glucocorticoids as compared to the baseline could indicate that the animal is in a negative state (e.g. fear), but the same effect would be observed if the animal is aroused positively and thus, in a positive state (e.g. reward anticipation). Without appropriate context, the elevated glucocorticoid level thus turns out to be an indicator for emotional arousal in general rather than indicating a negative state (Ralph and Tilbrook 2016). Play behavior, to take another example, is generally considered a good indicator of positive emotional states, but in some cases, increased play activity was connected with a negative emotional state of the tested animal as assessed by an independent method (Ahloy-Dallaire et al. 2018; Richter et al. 2016). Consequently, even commonly used indicators can fail to indicate the emotional state correctly when considered alone or taken out of biological context. Let us call this type of problem *the specificity problem* of emotional indicators, for the emotional indicators are not always specific in depicting unique emotional states.^3^

The second type of problem concerns the credibility of an indicator *as an indicator of any emotional state*. The observed physiological and behavioral changes are not exclusively caused by *emotional* states. For example, heart rate could be influenced by a metabolic change, without involving emotional states. Thus, heart rate may not be a credible indicator of emotional states in general. Let us call this type of problem *the credibility problem* of emotional indicators.

Notice that these two inherent problems of emotional indicators concern different levels. The specificity problem concerns the question of which one of all the possible emotions is indicated. The credibility problem concerns a question one level above the specificity problem, namely whether an emotion or something else is indicated. The two problems may occur distinct from each other or combined. An indicator could, for instance, be a very credible indicator of emotional states (meaning it always indicates *an* emotional state) without being specific about which emotional state it depicts. However, an indicator could also be highly specific in depicting exactly one unique emotion among the emotions without being credible (meaning it could also indicate something different from an emotion; it occurs sometimes without the emotion). Furthermore, specificity and credibility both come in degrees.

One way to overcome these epistemic difficulties is to look for new ways of assessing animal emotional states that are: (1) more emotion-specific; (2) more credible as indicators; or (3) give rise to more credible and/or emotion-specific indicators *in combination with* already existing indicators. A relatively new and popular emotional indicator that is assumed to overcome these problems is judgment bias. Before scrutinizing the explanatory power of judgment bias experiments, let us see exactly how these experiments are set up.

**Fig. 1:**
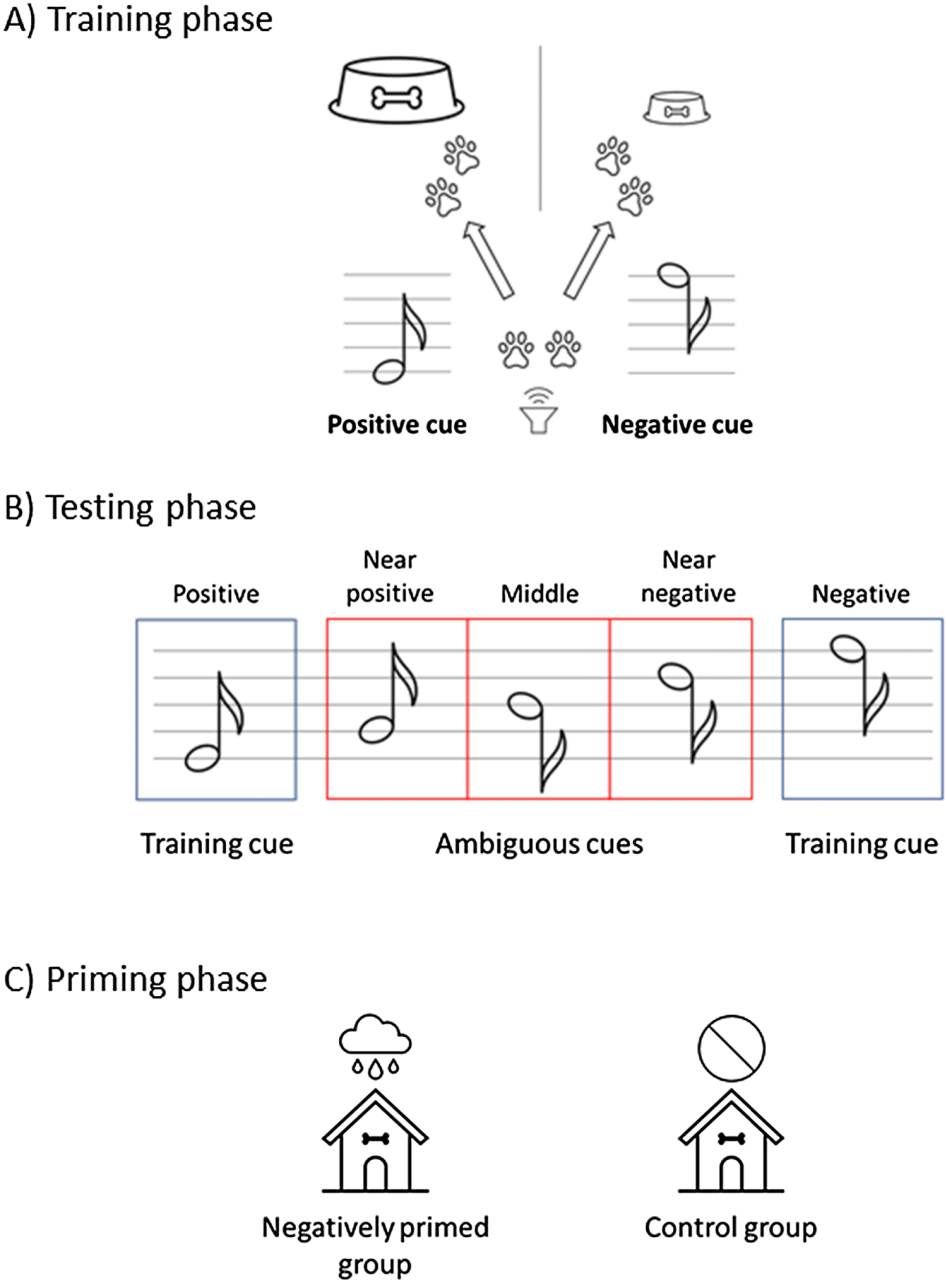
Example of judgment bias test design. **A** *Training phase*: When presented with a low-pitched tone, animals learn to move to a location on their left side to get a large reward (positive cue). In contrast, after a high-pitched tone, they learn to move to a location on their right side to get a small reward (negative cue). After satisfying certain learning criteria (e.g. 80% of correct responses) they proceed to the testing phase. **B** *Testing phase*: In addition to training cues, animals are also presented with tones with pitches intermediate to the training tones—three ambiguous cues. If an animal responds to an ambiguous cue by moving to the left side, it is interpreted as expecting the large reward; an “optimistic” response. However, if an animal responds by moving to the right-hand side, this is interpreted as expecting the small reward; a “pessimistic” response. **C** *Priming phase:* Before the testing phase, animals are split into two groups and one of the groups is exposed to unpredictable housing to induce a negative emotional state. The control group is unmanipulated

*Cognitive judgment bias test.* Judgment bias experiments were, among other things, designed to show that “decision-making”^4^ and judgment in non-human animals are influenced by emotional states. The design of judgment bias tests can be generalized as follows (see Fig. 1 for a simplified example, and Lagisz et al. 2020 for more details). Animals are first trained to respond differently to two distinct cues of the same perceptual dimension (e.g. visual, auditory, or spatial cues): when a “positive” training cue is presented, they obtain a reward; and when a “negative” training cue is presented, they receive a smaller reward or even a punishment. By repeated trial and error, they learn to receive the reward by responding in one way (e.g. move to a location on their left side after a low-pitched tone was played), and to avoid punishment (or to obtain a smaller reward) by responding in another way (e.g. move to a location on their right after a high-pitched tone was played).^5^ This is the *training phase* of the test (Fig. 1A).^6^

When the animals have learned to respond correctly to training cues, they proceed to the test where they are periodically presented with “ambiguous” cues, which are interspersed between training cues—this is the *testing phase* (Fig. 1B). Ambiguous cues are located qualitatively between the training cues associated with negative and positive effects, so that they belong to the positive and to the negative side at once—hence ambiguous. As with the training cues, only one ambiguous cue is presented at a time, but the responding animal is usually not rewarded or punished. The behavioral response to ambiguous cues is considered to indicate whether the animal “anticipates” positive outcomes (responding as if expecting a reward) or negative outcomes (responding as if expecting punishment or a smaller reward). These responses are shown to be sensitive to a change in emotional state (Lagisz et al. 2020; Neville et al. 2020) and they could be used as indicators of emotional states.^7^

For interpreting the behavioral responses to the ambiguous cue, it is required to know in which emotional state the animals are. Therefore, they are manipulated in a (emotionally) *priming phase* (Fig. 1C) to be in a certain emotional state before being tested (in the testing phase). A typical setting divides animals into two groups. One group is manipulated by a treatment considered to induce or change the emotional state. The other group serves as the control group and remains unmanipulated. To induce negative states, “negative” priming could be unpredictable housing, lasting throughout the training and testing phase of the experiment, or an enforced electrical shock applied just before the testing phase. Similarly, treatments considered to induce positive states can be used as “positive” priming (e.g. providing enrichment).

We may observe any of the following outcomes of such a judgment bias experiment with “negative” priming:

- 1. The animals from the “negatively” primed group may show a negative judgment bias by responding more often in the negative way with respect to the ambiguous cues, i.e., by the behavior they have learned to avoid punishment, than animals from the control group. The interpretation of this result would be that animals from the “negatively” primed group are in a negative emotional state and thus, that this particular priming is inducing a negative emotional state. The same goes for “positive” priming. Depending on the goal of the study, this would be considered as the validation of the testing paradigm (i.e., the test can detect changes in emotional states) or evidence that the used priming manipulation indeed changed the emotional state of the animal (e.g. tested enrichment induces positive emotions and thus increases welfare). Additionally, if not already presupposed, this finding would indicate the existence of “emotion-like” states in the tested species (e.g. Solvi et al. 2016).
- 2. The animals from the negatively primed group may show a positive judgment bias by responding more often in the positive way with respect to the ambiguous cues (and vice versa for a positively primed group). There is no definite interpretation of this result without any additional more detailed information about the animals, their cognitive abilities, and the contexts in which such behaviors may occur. These unexpected priming effects were reported in the literature: for example, adrenergic-system targeting drugs induced negative instead of positive bias (Neville et al. 2020) and stressful events induced positive instead of negative bias (Brydges et al. 2012).
- 3. Emotional priming may also not lead to any change in the interpretation of the ambiguous cue, so that there is no statistically significant difference between the treatment and the control groups. This may mean that the priming did not evoke any emotional state that lasts until the judgment bias test, or that the bias is too small to be detectable with a concrete experimental setting. However, as long as data from different individuals are averaged, it may also be the case that individuals react to the same treatment with different emotions, some with positive ones and others with negative ones.

Let us now come back to the epistemic problems with emotional indicators. It seems quite obvious that the judgment bias test inherits the credibility problem. The described experiments may show that there is a correlation between a treatment expected to induce certain emotional states and behavioral responses in a judgment bias test (as described in outcome 1). However, different factors, besides the emotional state, can influence judgment bias, such as feeding motivation^8^ (reviewed in Whittaker and Barker 2020). Or, as another example, treatment could improve learning, so that primed animals learn faster that the ambiguous cues are not rewarded and respond more as expecting a smaller reward—“pessimistically” (discussed in Mendl et al. 2009). Consequently, if it is possible and plausible that cases of judgment bias could occur without any involvement of emotional states, then judgment bias has the same credibility problem as other indicators. This, of course, does not mean that the overall credibility could not be increased if we took additional indicators into account. The point is rather that if we look at each emotional indicator (including judgment bias) separately and try to assess the emotional states by it, then the credibility problem remains unsolved.

At first glance, it seems that the specificity problem, too, accompanies judgment bias as an emotional indicator. It is hard to imagine that one could be able to specify the exact kind of emotional state of an animal (fear, anger, depression-like states, joy, frustration, etc.) just from the judgment bias, be it negative or positive. However, the experiments seem to suggest that there are correlations between negative bias and negative emotional states in general and between positive bias and positive emotional states in general (Lagisz et al. 2020). This is certainly a relevant differentiation and may, in many cases, even be sufficient from the particular perspective of animal welfare scientists, because, as mentioned before, their interest often is assessing whether or not animals are in negative (or positive) emotional states. Therefore while, for example, an elevated level of circulating glucocorticoids could indicate either a state of fear or one of excitement and thus does not allow inference of a negative or positive emotional state, a state of fear would usually correlate with a negative judgment bias and a state of excitement with a positive one. In this respect, judgment bias promises to be more specific than some other indicators.

To sum up, in light of inherent epistemic problems of traditional emotional indicators, there are at least two reasons to consider judgment bias tests as an alternative: (1) where emotional indicators have different degrees of credibility, having another indicator *in addition to* the already existing ones can increase the overall credibility of the set of indicators when all are pointing to the same emotional state; (2) with a cognitive bias test, it seems possible to assess whether a certain treatment *induces* a positive or negative emotional state, which is of eminent value for animal welfare science and biomedical science.

Having discussed some inherent problems with emotional indicators and established the epistemic motivation of judgment bias tests, let us now discuss the underlying cognitive mechanism.

## Underlying cognitive abilities

We are now going to scrutinize the cognitive abilities that could underlie and explain behavioral responses to ambiguous situations in judgment bias tests. Before introducing possible candidates, however, let us first specify the category of cognitive abilities. With this category, we are referring to mental (though not necessarily conscious) capacities, like the abilities to represent, to re-identify, to have emotions, to perceive, and also to higher level mental capacities like to have and use memories, to judge, to plan, to reason. Some of these capacities are more complex or more rudimentary than others and some presuppose each other. However, these are to be discerned from the neuronal activities and processes that form the basis of these capacities. Such fundamental processes cannot *as such* explain animal activities and behaviors in judgment bias tests; the concept of judgment bias focuses on representational states rather than on their neuronal basis. This can be seen in both “folk psychology” and empirical sciences. Consider, for example, answers to questions like: “Why is that squirrel climbing that tree so fast? Why is that honeybee flying in that direction?” The answers would usually refer to representational states or abilities rather than to—unknown—neuronal states: because the squirrel is *scared of* and *running away from* the dog (representing it *as dangerous*) or because the honeybee *represents* the nectar to be in that direction, say, as a result of *observing* the dance of a fellow bee and *interpreting* it (possibly non-consciously) *as representing* the nectar occurrence in that direction. Analogically, the answer to the question of why the animals in the cognitive bias tests respond to the ambiguous cue in a specific way would refer to some kinds of emotional or inner representational states or abilities. That is why ascertaining possible representational abilities that can result in the behaviors in question has explanatory value for scientists conducting cognitive bias tests.

A last remark before we analyze the underlying mechanisms: this is not an analysis of the terms “ambiguous,” “bias,” or “judgment” or of their applications. Our listed candidates of inner states and abilities that explain the reaction to the ambiguous cue in the judgment bias tests may or may not confirm the usage of these terms—whatever the criteria of this confirmation may be. Nevertheless, our focus is not on this kind of confirmation but on plausible candidates for different cognitive abilities that would result in similar behavioral outputs with similar input conditions in these tests.

### Plausible candidates

Scientists experimenting on judgment bias often do not ask the question about the (exact) kind of cognitive abilities that bring the bias about. They are very cautious, classifying the responses as merely being “as if” the animal expected a certain outcome (Mendl et al. 2009; Paul et al. 2005; Roelofs et al. 2016). Usually, they treat the involved cognitive mechanism as a black box and track it through its behavioral outputs.^9^ As clarified before, we think that this question is worth answering from both perspectives, that of cognitive science and that of animal welfare science, as it could lead to refined measurements, development of new tests, and a better understanding of emotional states in general.

Our approach to answering the above question is to make a list of cognitive abilities that are discussed in philosophy of cognition and that we, at the same time, consider as being evolutionarily plausible candidates that may produce the biased output in a systematic or regular way. This will outline some of the possible and plausible underlying abilities that contribute to the mechanism in the assumed black box. The answer would in part require describing some inner states of the animals *as* representing the external cues, i.e., assuming that the states are *directed at*, or *are about* an external phenomenon or state of affairs (e.g. Sterelny 1990).

In our list of plausible candidates, we mostly focus on representational capacities. This does not mean that there are no other cognitive abilities involved and/or relevant for the judgment bias tests. For example, one of the most central feats of the cognitive system is to use information gathered in previous encounters with an entity in the current or future encounters with that entity (Millikan 2000). This important ability is usually referred to as the ability to generalize. This ability is of utmost importance in dealing with situations that are similar to, but not identical to known situations. Generalization, therefore, has immense selective advantages (Cheng 2001; Shepard 1987).^10^ The evolutionary impact of the cognitive act of generalization can, among other things, be used to explain animals’ learning ability in general. In fact, since the judgment bias test is based on the animals’ ability to learn training cues in the training phase, there would be no judgment bias test without animals’ ability to generalize.^11^ Another example is animals’ ability to have and use memories. Without it, there would be no learning. Therefore, when we are talking about looking into the black box of underlying cognitive abilities in these tests, we are not talking about *all* the underlying cognitive abilities that are involved. Our focus in this paper is animals’ *representational* ability. More specifically, we want primarily to scrutinize what animals perceive/represent when they are confronted with the ambiguous cues.

*1. Constitutive lack of discrimination*. It is plausible that the cognitive system of some animals does not discriminate between the cue that, *from our perspective*, should be ambiguous for them, and one of the training cues. This inability may be a “constitutive” lack of discrimination between ambiguous and training cues, and would not be mediated or altered by emotional states and other conditions, for it is a matter of physiology and unmodifiable by priming. Imagine, for example, somebody who suffers from a particular kind of color blindness and cannot discriminate between, say, blue and purple but can distinguish red. This person now receives a purple cue, meant by the experimenter as a middle cue between blue and red, and sees it as blue. The test person’s perceptual apparatus simply does not discriminate between what we would classify as a middle cue and as one of the others. Now imagine that this is the “normal” case for the whole species that is being experimented on; the cue would not be ambiguous for individuals belonging to this species.

This possibility is eliminated if animals show the ability to discriminate between the cues in a separate experiment or if animals respond differently to ambiguous cues than to the training cues in the judgment bias test. This seems to be the case in most published studies since different responses to at least some ambiguous cues are considered a prerequisite for a valid judgment bias test (Gygax 2014, 61). We are mentioning this case for reasons of completeness, and also because it helps to better understand the other candidates.

*2. Misrepresentation*. One of the most plausible situations that may hold is that the ambiguous cue is represented—wrongly—as one of the cues the animal was trained upon, i.e., that it is misrepresented (cf. Dretske 1986; Godfrey-Smith 1989; Neander 1995). Assume the cues trained upon were squares and circles, and the ambiguous cue was an octagon. If the content of the representation is an octagon (howsoever one could possibly find this out), the ambiguous cue would be represented correctly. If the ambiguous cue is represented either as a circle or as a square, or in the very way a circle or a square is represented, it is misrepresented. Or consider this standard example of a misrepresentation: a frog or a toad reacts to almost any moving object with appropriate color, size, and shape relative to the direction of its movement by certain prey-catching behaviors (Ewert 2004; Lettvin et al. 1959). It represents the object as, say, a nutritious flying prey. If the object is actually a fly the representation is true. However, if it is a non-prey black particle, let us say a small moving black piece of paper in the air, and the prey-capture mechanism of the frog triggers a tongue-dart in the appropriate direction and captures the piece of paper, the frog/toad misrepresents that object. That is, the piece of paper is not represented as a piece of paper (which would be impossible as long as we assume that this category does not exist at all for the frog). Instead, if represented at all, it is represented as something else with which the frog/toad is familiar—in this case as a fly, which results in triggering the tongue-dart.

Mendl et al. mention that a misrepresentation of an ambiguous cue as a “familiar one” may be the case with ambiguous cues that are very similar to training cues but argue that this is likely not to be the case when ambiguous cues can be easily distinguished from training cues (Mendl et al. 2009, 172).^12^ Therefore, to exclude misrepresentation, does one simply need to confirm that animals can discriminate between ambiguous and training cues in classical discrimination experiments where two cues are presented simultaneously? This would be too quick a conclusion. Consider the following: just because one is able to distinguish between cats and dogs under ideal or standard conditions, it does not mean that one is not likely to confuse them under certain circumstances or in certain contexts, e.g. to mistake in dim light a small dog for a cat. Similarly, just because animals showed the ability to discriminate between the ambiguous cues and the training cues, they need not be able to do so under testing conditions of judgment bias experiments, where multiple ambiguous and training cues are presented sequentially with time gaps in-between (as mentioned by Mendl et al. 2009, 173). They may still misrepresent ambiguous cues as one of the training cues. Misrepresentation can occur for various reasons. The reward is just too delicious, or at least delicious enough to mistake anything *resembling* the positive cue as *being* the positive cue; or the punishment is too severe or severe enough so that anything resembling the negative cue gets mistaken as being the negative cue; or the emotional inducing phase made the test animals too cautious, too afraid, too anxious, too bored etc.

**Fig. 2:**
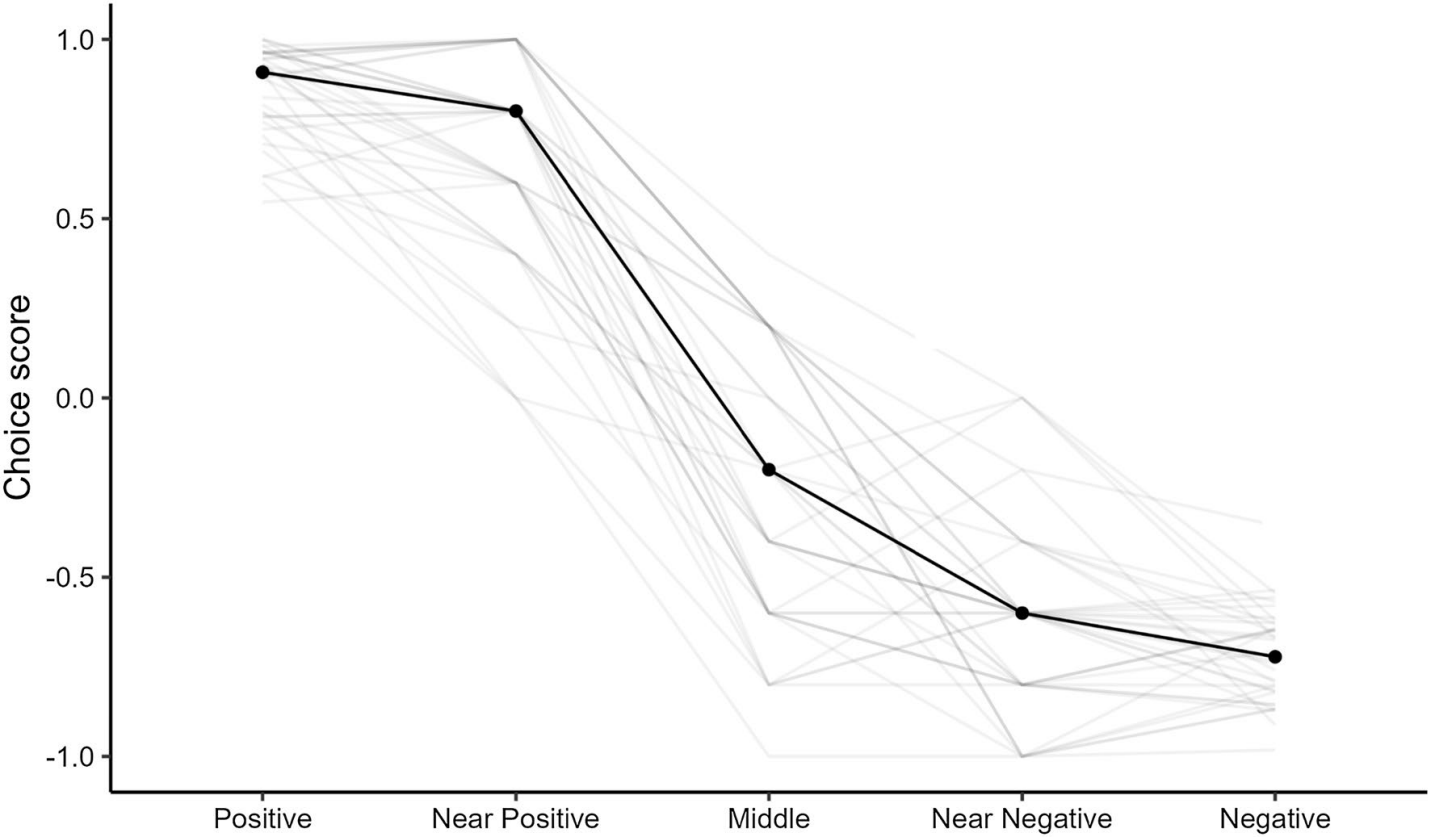
Empirical example of a reaction toward cues in a judgment bias test resulting in a monotonic response curve. Data are obtained from a judgment bias test in mice based on visual cues (Bračić et al. 2022). The choice score indicates the proportion of responses where mice chose to respond as if expecting a positive outcome compared to choices as if expecting a negative outcome (close to + 1 = more “optimistic” choices, closer to − 1 = more “pessimistic” choices). The score is based on 10 responses for each ambiguous cue and 120 for each training cue. Data are presented as the median score of all individuals for each ambiguous cue (point), connected with lines to represent the response curve. Shaded lines represent the response curves of each individual. Number of individuals: *N* = 39

As an argument for the decision-making ability, Mendl et al. use the observations that there is a gradual change in response to cues in judgment bias tests (Mendl et al. 2009, 173). In a typical judgment bias test, animals are often introduced to three ambiguous cues; one ambiguous cue is closer to the positive (near-positive), one is closer to the negative training cue (near-negative), and one is perceptually in the middle. This scheme is applied to test whether there is a gradual change in animals’ responses across the cues. For example, animals reduce lever pressing from the positive cue via ambiguous cues to the negative cue, thus producing a monotonic response curve (for an empirical example see Fig. 2). If there is a gradual change in responses, it is presumed as validating that animals interpret ambiguous cues in reference to the training cues (e.g. Gygax 2014, 61; Hintze et al. 2018, 10). Assuming that the middle ambiguous cue is not perceived as actually being one or the other of the training cues, Mendl et al. consider that it is likely that something like decision-making is happening. Although we grant that something like this may be happening in animals with higher cognitive abilities, which we will consider next, we want to emphasize misrepresentation as being one of the most likely scenarios, even in cases where one may consider decision-making as being an alternative mechanism. To be clear, our estimation of likelihood here is not based on empirical data but rather on the principle of Ockham’s razor to be as scarce as possible with assuming entities, in this case with presupposing involved cognitive instances or abilities. In fact, if it is likely that the animals are misrepresenting the ambiguous near-positive and near-negative cues, we do not need to—and should not—bring some more complex^13^ cognitive abilities, like decision-making, into play to explain the response to the middle ambiguous cue as long as there is no concrete indication for involvement of the higher capacity. In the case mentioned, there is no such independent argument for the presumption that the animals’ gradual responses indicate decision-making. What we want to get across is that as long as the subject animals react to the ambiguous middle cue, say, as they would to the trained positive cue, they misrepresent the cue. It is not (or very unlikely) that they misrepresent the ambiguous near-positive as being positive and represent the ambiguous middle as being a middle sign between two learned cues and then “decide” to go for positive *just in case*. What we want to emphasize is that although there may be a gradual shift in responses, it still does not mean that there is no misrepresentation. However, *how* exactly the animals misrepresent the ambiguous cues could, of course, differ in each case (there could be different levels of information processing involved). What exactly the gradual shift in the responses indicates is another question and will depend on the species. There could be some learning processes taking place and so on. If at some point, the animals stop reacting to the ambiguous cues, that would mean that they no longer misrepresent them as training cues.

Nevertheless, misrepresenting is not decision-making and because it is possible that more complex cognitive abilities would produce a similar output under similar input conditions (as in the case of humans), we will still consider this option and try to identify the minimal requirements of such a cognitive system according to an evolutionary perspective.

*3. Conflicting content(s)*. The third possibility which could be available in an advanced cognitive system is the representation of the ambiguous cues *as* ambiguous, for example, as something undetermined between two or more *specific* states or objects. To have an analogy from the perspective of a (human) viewer, it is *not* like: “I am seeing something but I don’t have any idea what it is,” but more like “I am seeing something that is either *x* or *y*, but I cannot exactly tell which one of those two.” The latter is analogous to the cases that we are considering now.

It is important to note that the conflicting content(s) could be different contents of different representations of the same state of affairs, or a “conflicting” content of a single representation of that state of affairs. Without going too deep into the theories of content, with a *conflicting* content of one representation we are referring to a content that has two or more aspects with different psychological roles (hence “conflicting”), e.g. a state of affairs is represented as being a dog or a cat or even as a dog or a non-dog, where there are different behaviors associated with these different aspects, for example fleeing in case of the representation of a dog and attacking in case of a cat or a non-dog. How exactly these aspects are represented and how the connections between them appear is not relevant here. It is merely relevant that the cognitive system links these different aspects to different behavioral outputs^14^ and that the cognitive system has the means to deal with this conflict.

While it may sound natural that humans have such representations, the issue is much more complex than it appears at first glance. In general, the state of affairs in question needs to be represented *as* conflicting (either through the conflicting representations or the conflicting aspects of a representation of the state of affairs), which furthermore means that there are mechanisms, over and above “regular” representational mechanisms, that evaluate these representations and compute, or “decide” about, the generation of an output signal that enters the behavior-producing mechanisms. This feat of the cognitive system is a capacity over and above the ability to represent (and misrepresent) something in a specific way, because simple representational systems do not usually evaluate representations or aspects of a representation *against each other*.

We want to emphasize that we are not suggesting that there is no evaluation of representations or some kind of computing happening in cases of mere misrepresentations. However, if the animal has a representation with a conflicting content or competing representations, then some kind of *resolving*-mechanism should come into play that deals with the ambiguity. Doing this in a consistent way requires the involvement of a different, more advanced cognitive ability than would be required in reacting to a mere misrepresentation of a cue. Bear in mind that from the setup of the judgment bias experiments there is not yet much known that allows us to assess which kind of these cognitive abilities (misrepresentation *versus* conflicting contents) are in play. Our analysis suggests a way of gaining better knowledge about the representational systems, i.e., a way to open the black box at least a little bit; does the animal always react the same way to an ambiguous cue, or does it learn to distinguish it from the training cues? One might expect that conflicting content is interpreted cautiously or with hesitation on the first confrontation, but more decisively in later ones, while a plain misrepresentation would give rise to less hesitations.

A judgment bias test, however, would merely hint at certain mechanisms and cannot be used to conclusively distinguish between cases of misrepresentation and of conflicting contents. We will therefore discuss, in “Ways of differentiating: a new proposal”, more complex experimental setups that could yield more definite results on the representation mechanism involved.

*4. Novel representation*. The last option that we want to consider is the possibility of having a *novel* representation, i.e., to represent the state of affairs—the ambiguous cue—*as novel*. To use the analogy from before, it is more like: “I am seeing something but I don’t know what exactly it is.”

Representing something as novel does not mean that the representation is marked by a “novel”-index. It also does not mean that the one having the representation “thinks” the content is novel (conscious or not). All it means in this context is that the one having the representation has not yet gathered any prior information about what is being represented, which includes in particular that it does not relate the novel representation as being related to the training cues. As mentioned before, one of the most central feats of the cognitive system is to use information gathered in prior encounters with an entity in the current or future encounters with that entity. It is, therefore, common for the cognitive system to start tracking and gathering information about newly encountered unknown entities.

Representing ambiguous cues as novel is more likely in certain types of judgment bias tests. The most prominent case is when the cues do not differ in only one perceptual dimension (e.g. Douglas et al. 2012; Nogueira et al. 2015; Salmeto et al. 2011). For example, Douglas et al. (2012) used different acoustic sounds: a note on a glockenspiel and a dog-training clicker as training cues, and a squeak from a dog toy as the cue that was considered ambiguous. However, do animals perceive these sounds to be different in frequency, noise level, or some other dimension? In such cases, it is not clear how animals relate ambiguous cues to training cues; they could be represented as novel (as mentioned by Roelofs et al. 2016). Although for a different reason, novelty could also play a role in judgment bias tests that are based on spatial cues. In this type of test, ambiguous cues are represented by a novel location which is in-between the training cues (e.g. Briefer and McElligott 2013; Richter et al. 2012). Jardim et al. (2021) showed that, in this design, reaction to the ambiguous situation depends on how explorative an individual is and thus, includes the animal’s response to novelty. As individuals (and species) consistently differ in their response to novelty, this may lead to misinterpretation of group differences or treatment effects from judgment bias tests involving novelty (Jardim et al. 2021). As seen from these examples, it is possible that, at least in some judgment bias tests, animals represent situations as novel that are intended to be ambiguous.

How an animal will respond if it represents an ambiguous cue as novel depends on various factors, such as the level of individual development of the cognitive system, the individual’s prior learning experiences, the overall cognitive capacities of the species, the organism’s predispositions, and of course the organism’s present environment and emotional state. However, if the organism had a genuinely novel representation, it could be expected that it would change its behavior depending on the kinds of information being gathered about the entities in question (here, the ambiguous cue). For example, in judgment bias tests where there are multiple presentations of ambiguous cues and they are not associated with any reward or punishment, the animal could quickly start to ignore these cues. This would suggest that (at some point) the animal has had a novel representation of the ambiguous cue and that the representation has a different content than the representations of the previously learned ones. Indeed, similar cases were observed in many studies and it is discussed in the literature as “loss of ambiguity” or “extinction of response” (reviewed in Roelofs et al. 2016; Whittaker and Barker 2020). Individuals or treatment groups could differ in the speed of “loss of ambiguity”, due to differences in learning abilities, which would lead to spurious association with the test results.

In this section, we identified *misrepresentation*, *conflicting content*, and *novel representation* as potential underlying cognitive abilities in judgment bias tests. However, before describing our suggestion about (practical) ways of differentiating these options, let us make some important clarifications. First, we do not suggest that our list of possible candidates for mechanisms is complete. This is the list of options that we think are the most plausible candidates for the underlying representational abilities. Others may be possible. Second and most importantly, we do not think that these possibilities are mutually exclusive. In other words, it is possible that the underlying cognitive ability of a process studied is a complex combination of these options. For example, an animal could misrepresent the ambiguous cue at first but start perceiving it as novel later and change/adjust its behavior accordingly; or the animal could perceive the cue as novel but misrepresent some aspects of it as being dangerous or advantageous and so on. This means that assessing the exact configuration of the underlying cognitive mechanisms through experiments requires thorough planning, more complex training phases (we will address this in the next section), and various controlling scenarios, which taken together may be near impossible to conduct for some species. Nevertheless, in the next section, we will suggest a setting that is less likely to involve *misrepresentation*.

### Ways of differentiating: a new proposal

As we stated earlier, the possibility that the tested animals may lack the ability to discriminate between the ambiguous cues and the cues in the training phase can be eliminated through separate experiments that test their perceptual abilities. However, things are more complicated if we are to establish whether a behavioral output of the judgment bias test is the result of a *misrepresentation*, of *conflicting contents*, or an instance of *novel representation*.

To eliminate one candidate mechanism that may bring bias about, namely misrepresentation, we propose using a setting that involves a combination of positive and negative cues in the testing phases.^15^ Any bias found with this setting will more likely be due to *conflicting contents*.

**Fig. 3:**
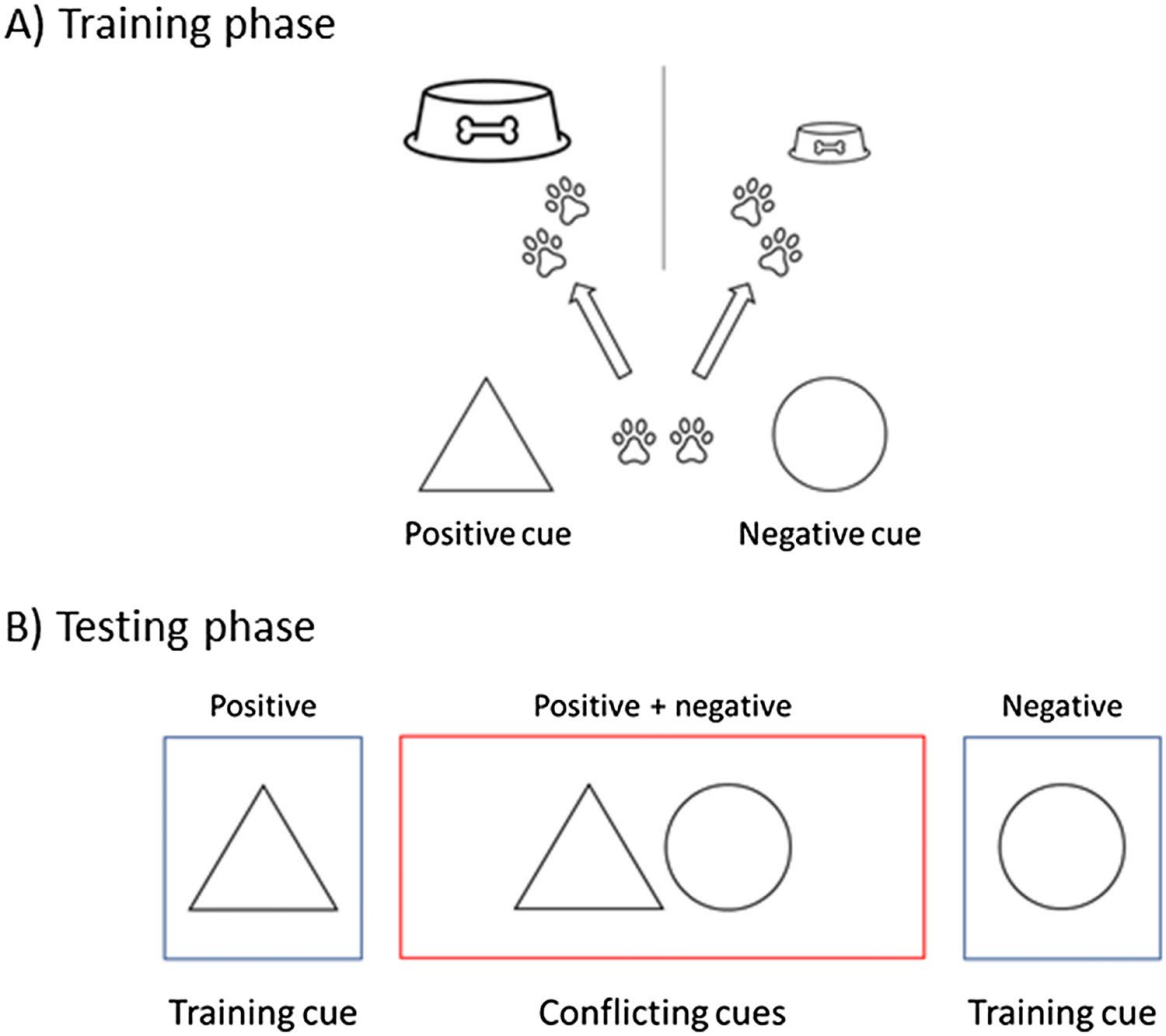
Test setting focusing on conflicting content with one sensory mode. **A** *Training phase*: When presented with a triangle, animals learn to move to a location on their left side to get a large reward (positive cue). In contrast, they learn to move after the presentation of a circle to a location on their right side to get a small reward (negative cue). After satisfying certain learning criteria (e.g. 80% correct response), they proceed to the testing phase. **B** *Testing phase*: Animals are presented with both the positive and the negative training cues simultaneously—conflicting cues

The *training phase* is similar to that in the judgment bias test: animals learn to respond in one way to the positive cue—associated with a positive outcome—and in another to the negative cue—associated with a more negative outcome (Fig. 3A). However, in the *testing phase*, animals are exposed only to those training cues rather than using novel cues that are supposed to be ambiguous. Specifically, positive and negative cues are combined so that the animal is simultaneously presented with both training cues (Fig. 3B). Therefore, in contrast to a judgment bias test, the “ambiguity” is represented not by one intermediate cue, but rather by two different, conflicting cues.

To reduce the probability of cues overriding each other (e.g. animals just focusing on the negative cue), we propose to even address different sensory modes (e.g. visual and auditory).^16^ In the training phase of such experiments, animals need to learn to associate two different sensory cues^17^ with negative and two with positive outcomes. It is important to test both options of conflicting cues, a positive cue 1 with a negative cue 2 and a negative cue 1 with a positive cue 2. This makes it less likely that one of the cues generally overrides the other.^18^ Individuals would need to provide relatively consistent answers to both conflicting cues for the experiment to be valid. At the cost of longer and more complex training and testing phases, one could even test an animal’s response to different combinations of different numbers of positive and negative cues (e.g. two positive cues and one negative cue). With this design, it would be possible to test whether or not there would be a gradual response similar to that shown in Fig. 2.

**Fig. 4:**
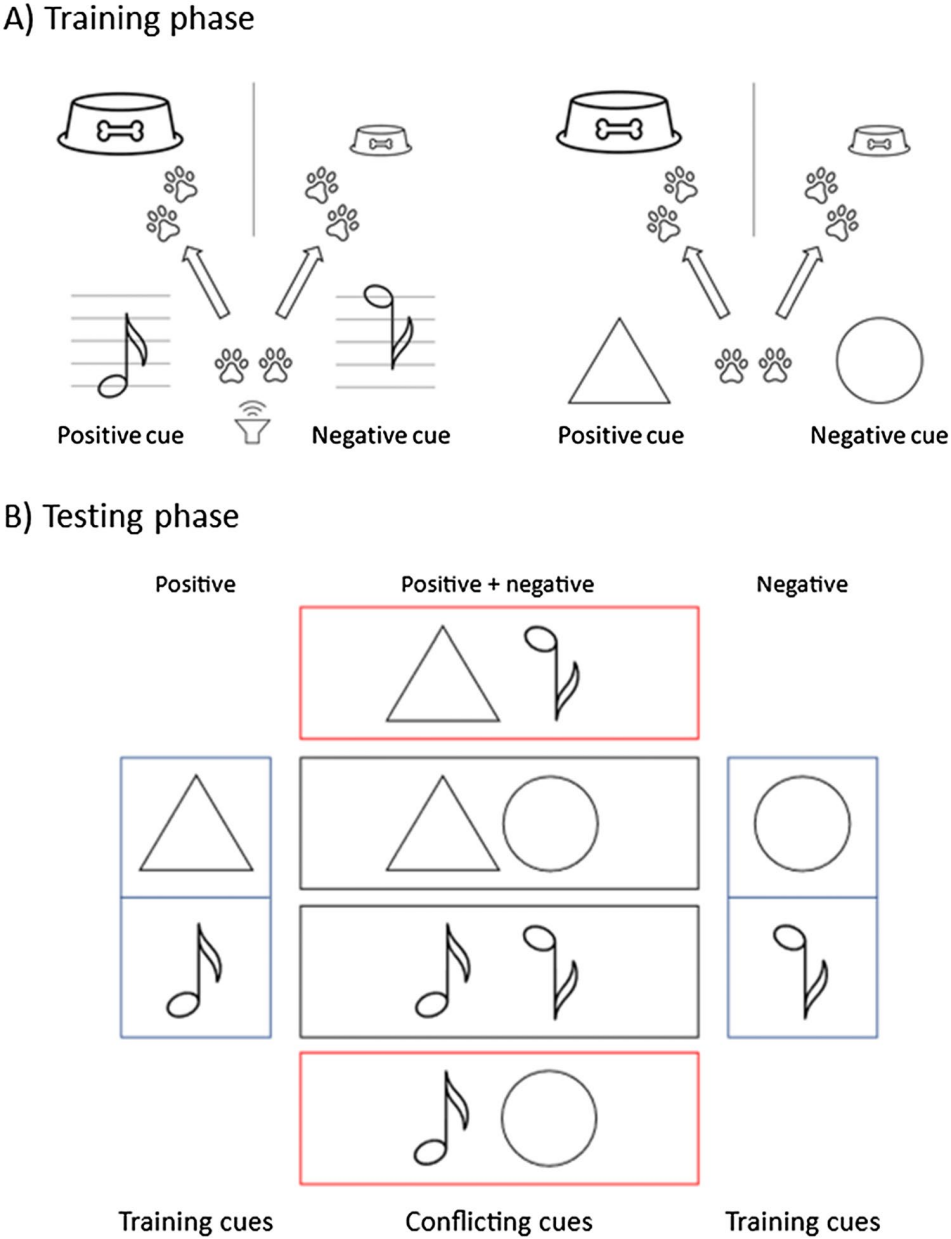
Test setting focusing on conflicting content with two sensory modes (auditory and visual). **A** *Training phase* (blue rectangles): When presented with a triangle or a low-pitched tone, animals learn to move to a location on their left side to get a large reward (positive cues). In contrast, after the presentation of a circle or a high-pitched tone, they learn to move to a location on their right side to get a small reward (negative cues). After satisfying certain learning criteria for all the cues (e.g. 80% correct response), they proceed to the testing phase. **B** *Testing phase*: As in the setting with one sensory mode, animals are simultaneously presented with both a positive and a negative training cue (conflicting cues), but the conflicts now also involve cues over different sensory modes (red rectangles)

We want to emphasize two points here. First, it is possible for subject animals to still misrepresent the conflicting cues in our setting as being either positive or negative, or for animals to have a novel representation of the compound cues. However, because the middle cues in our setting now contain the learned cues of the training phase, consistent answers to them would more likely indicate the animals’ ability to resolve conflicting content. Random/inconsistent answers of the individual would suggest a lack of relevant mechanisms of resolving conflicting contents.

Second, this experiment is not supposed to solve the problems of the judgment bias test and to replace it. The suggested setup serves a different purpose, namely singling out specific mechanisms underlying the judgment bias, in the case of scientific interest in doing so. This setup is primarily supposed to test whether animals possess certain mechanisms to resolve conflicting contents.

*Possible outcomes*. In the following, we discuss the possible outcomes of such an experiment and show which conclusions could be drawn with respect to how the underlying cognitive system represents the cues.

The individuals are trained to the cues and then exposed to ambiguous combinations of cues, without any prior exposition to emotion-eliciting conditions. Let us assume that the punishment and reward in the experiment are “fair,” i.e., they are not too highly evaluated by the animals.^19^

- A. Each individual may show a consistently biased answer, positively in some individuals and negatively in others. This would allow ascription of a dispositional trait to the individuals that counts as long-lasting. Relative to other tested individuals, we could call these individuals “optimistic” or “pessimistic” decision-makers.
- B. All individuals may show a similar bias, either positive or negative. One could interpret this as constitutive optimism or pessimism being a certain dispositional trait of the species under investigation, where either a positive or a negative cue overrides an opposing cue. The existence of such “optimistic” or “pessimistic” species traits may be expected if they were selected for due to certain living conditions.^20^
- C. The answer may be found to be arbitrary in all individuals, i.e., the ambiguous combination of cues leads to positive and negative answers in statistically indiscernible proportions in each individual. The conflicting contents, which in isolation lead to a positive and negative answer, respectively, level out. This outcome would strongly suggest that the animals do not possess the relevant mechanisms of resolving conflicting contents^21^ for this kind of situation (though it does not rule out that a modified or refined experiment may indicate the presence of other mechanisms of resolving conflicting contents, e.g. one using different cues or cues of different intensity).

It is, however, important to point out that using compound cues could have unintended consequences which could influence the results. For example, it is possible that confronting the compound cues would be emotion-inducing by itself, e.g. the animals get frustrated or excited as a result of perceiving the compound cues, which on its part could affect their behaviors. This could be the case on the individual level and elicit consistent optimistic or pessimistic behavior of an individual, with different individuals reacting differently (A); or it could happen on the species level, producing consistent (either) optimistic or pessimistic behavior in all individuals (B).^22^ If such a feedback loop occurs, the experiment fails to single out (with appropriate certainty) relevant mechanisms for resolving conflicting contents, because the behavioral outcomes could be *solely* the result of emotional states circumventing “problem solving” at the representational level.

*Comparing the results with the judgment bias test.* If the proposed setting would result in something like (A) and the same kind of animal (i.e., another individual of the same species, or, e.g. of the same caste, social status, or developmental stage) would also show judgment bias in the judgment bias test, that would still not mean that the animals do not misrepresent the ambiguous cue in the judgment bias test. In fact, Parker et al. (2014) show that there is a positive correlation between the reactions of rats to a single ambiguous middle cue and to the conflicting cues, respectively. Although the behavioral response in both settings may be similar, it must not be inferred from the new setting, in which animals are confronted with a combination of unambiguous cues, that the explanation gained from this latter setting holds also for the first one. There is still no independent reason to assume that the animal would not misrepresent the ambiguous cue in the judgment bias test. It would, however, imply that for this kind of animal it is *possible* not to misrepresent the ambiguous cue and to represent it as conflicting. However, being possible is different from being probable. For assessing how probable this possibility is, other kinds of experiments and settings would be needed. On the other hand, if the result would be something like (C), where animals of the same species show judgment bias in the judgment bias test, then this would strongly suggest that the animals in the judgment bias test are misrepresenting the ambiguous cue. This could be the case because (C) would suggest that it is implausible to assume that the animals possess relevant mechanisms of resolving conflicting contents (otherwise there would be some kind of consistency in their response to the compound cues), whereas judgment bias would imply consistency in response. This output behavior would be explainable by misrepresentation. The reverse, however, does not hold. If somehow we knew that the animals are misrepresenting the ambiguous cue in the judgment bias test, it still would not necessarily mean, in our setting, that they do not possess the relevant mechanisms of resolving conflicting contents, for, as mentioned, misrepresentation can occur for various reasons. Consider this: we (human animals) might misrepresent a dog as a cat under some circumstances. Does it mean that we could not have, under other circumstances, a conflicting representation of something that is either a dog or a cat? Analogically, it is possible for animals to possess the relevant mechanisms of resolving conflicting contents but still misrepresent the ambiguous cues in the judgment bias test.

## Conclusion

Judgment bias tests allow the assessment of emotional states of non-human animals. In the first part of the paper, we discussed two inherent problems with emotional indicators: specificity and credibility. As these problems can potentially limit the epistemic value of emotional indicators, we suggest that every study using them should consider whether a particular measure specifically indicates only the emotional state aimed at, and how credible that measure is in indicating only emotional states and no other, non-emotional ones. In light of these problems, we argued for the relative epistemic preeminence of the judgment bias test as a relatively new way of assessing emotional states.

Central to judgment bias tests is confronting animals with ambiguous cues that are intermediates between cues they have learned to link to positive and negative consequences, respectively, and to act accordingly. The representational mechanism of decision-making in these tests is usually taken to be a black box. We discussed how this black box could be opened, at least a little bit, by considering representational abilities of the subject animals in these tests. Drawing on the philosophical perspective of understanding decision-making as a capacity of certain representational systems, we determined three different ways that ambiguous stimuli could in principle be represented: *misrepresentation*, *conflicting content*, and *novel representation*. We judge misrepresentation to be the most likely scenario. Misrepresentation, however, does not imply the involvement of more complex cognitive abilities that evaluate representations against each other. We propose a test regime in which the ambiguous stimulus is replaced by an ambiguous pair of unambiguous stimuli. This test regime makes it less likely that the animals misrepresent the ambiguous situation and aims primarily at testing the involvement of certain problem solving mechanisms that resolve a representation with a conflicting content. Finding out which species have this kind of mechanism would not only be an interesting result in itself, but could also help to better understand the mechanism of biased judgment in non-human animals, which could help further develop judgment bias tests. For example, if one is interested in attributing *decision-making* about ambiguous cues to subject animals, one may want to be sure that animals are not misrepresenting the ambiguous cues.

## Footnotes

1 What exactly the housing conditions were and what is meant by responding “more negatively to ambiguous situations” will be clarified and discussed in “Epistemic limits of emotional indicators”.

2 Note that those emotional states can still have unique patterns in other measures of physiology, behavior, or neural activity. Thus, we are not claiming that there are no reliable differences between (some) emotional states. This topic is connected to different views on the structure of emotional states, in particular between the theories of constructed emotions and basic emotion theories (for details see e.g. Barrett et al. 2007; Panksepp 2007; Scarantino et al. 2021).

3 The term “specificity” as used here is a *terminus technicus* from philosophical discussions on emotional states (e.g. Levenson 2003; Kreibig 2010). It is not the term used in behavioral biology or psychology.

4 We assume “decision-making” as not necessarily being a conscious process.

5 The association of a specific cue (e.g. tone pitch) with a large or small reward is usually counterbalanced. Also, there are test designs based on so-called go/no-go tasks where animals need to learn not to respond to “negative” cues, but this design suffers from a potential confounding effect of motivation to respond (for details on different test designs see Lagisz et al. 2020).

6 For interpretations of the judgment bias test in a Bayesian framework please see e.g. Trimmer et al. (2011). Training can be described as learning by Bayesian updating. The outcome of each trial modifies the expectation of the animal about the outcome of a certain reaction in a certain situation. In the beginning, it will have no or no strong expectations about the outcome. It will, however, have expectations when repeating the trial, and these will stabilize more and more with each consistent output to a reaction. The animal is said to have learned the task when its reactions are adequate and stable (e.g. 80% of correct responses).

7 If the ambiguous cue is directly in the middle of the two training cues, an organism’s Bayesian expectations with respect to a positive and a negative outcome should be equal. This means that the responses should be distributed randomly. Deviation from a random output could be then considered as internal bias of the animal. However, assuming a cue was “in the middle” can only be based on how the experimenters perceive the cues. Construing ambiguity in a combinatorial way, as we propose in “Ways of differentiating: A new proposal”, could in principle overcome this precariousness, but only if the mechanism is based on one of the three options of dealing with the cues we discuss in “Plausible candidates”, namely *conflicting content*.

8 Motivation for food reward is usually not viewed as an emotional state per se, but as a cofounding effect influencing other emotional states (Lecorps et al. 2021). However, declaring that the state of being hungry is not an emotional state is dependent on which emotion theory one subscribes to and should be argued for.

9 Mendl et al. (2009) sketched a picture of what they hypothesized as underlying mechanisms of judgment bias, which we will in part discuss in this section. However, they admitted that this may not concern animal welfare studies in practice: “From a practical animal welfare perspective it is perhaps not necessary to understand the processes underlying judgment biases” (ibid. 172).

10 While Shepard (1987) scrutinizes the relevance of the act of generalization in his attempt to find or define universal laws governing stimulus-response-relations in psychological science, Millikan (2000, 2017) tries to explain the biological function of the ability of practical knowledge and the ability to use/have empirical concepts. Nevertheless, both agree that this feat of the cognitive system is advantageous to the organism bearing it and that it has been shaped over evolutionary history.

11 For a discussion on the role of generalization in judgment bias tests see Roelofs et al. (2016).

12 While the mechanism of misrepresentation may be the same in the judgment bias test as in the case of the fly detection mechanism, its origin differs. In frogs, the detection mechanisms evolved as this particular detector that works well in a specific environment, but not necessarily in a novel environment. It is a “matched filter” in the sense of Wehner (1987). In the judgment bias test, the “filter” is established by individual learning rather than being matched by evolution.

13 We judge “decision-making” as a more complex ability relative to the mere ability to represent/misrepresent.

14 “Behavioral output” is to be understood in a broad sense and does not need to be a behavior of the animal in the strict sense. It includes, for instance, activities of some subsystems that are triggered by the representation(s).

15 This setting has been used in Parker et al. (2014) for a different purpose than we are proposing here. The authors suggested using it as a measure of attention bias. However, Crump et al. (2018) question why it should be considered more as attention bias than any other judgment bias test based on separate presentation of ambiguous cues.

16 Of course there should be several control groups with negative–negative, positive–positive, and negative–positive combinations with a different timely distance between the sensorially different cues.

17 Optimally, both senses should have similar perceptual values for the animals to avoid the possibility that the behavioral outcome is the result of the animals being overly sensitive to one of the cues.

18 Optimally, the test should also include conflicting cues of the same sensory modes (positive cue 1 and negative cue 1, and positive cue 2 and negative cue 2) as a control group (Fig. 4B).

19 Finding out whether or not the reward and punishment are evaluated “fairly” by the animals would involve prior experience and experiments which are designed specifically for the studied species.

20 The same outcome would be expectable if the punishment or reward are evaluated too highly by the animals. Alternatively, this result could simply reveal species-specific preference for some of the used cues (because of their shape and/or dimension). These options need to be excluded by proper test design.

21 There still may be mechanisms merely at the neuronal level to prevent “cognitive-freezing” and to cause the animals to get past the situation.

22 Special thanks to the anonymous reviewer for pointing out this important interaction.
